# Supplementary material for: β-lactamase expression induces collateral sensitivity in Escherichia coli
Source: Nat Commun. 2024 Jun 3;15:4731. doi: 10.1038/s41467-024-49122-2 (PMC11148083; doi:10.1038/s41467-024-49122-2)
Supplement: Supplementary file 3 — Description of Additional Supplementary Files [file 41467_2024_49122_MOESM3_ESM.pdf]

<u>Description of Additional Supplementary Files</u>

Supplementary Data 1. Plasmids used in this study.

Supplementary Data 2. Antibiotic resistance and genomic features of ECOR strains.

Supplementary Data 3. Statistical analysis of ATLAS surveillance database.

Supplementary Data 4. Antibiotics used in this study.

Supplementary Data 5. Oligonucleotides used in this work.
