# Supplementary material for: β-lactamase expression induces collateral sensitivity in Escherichia coli
Source: Nat Commun. 2024 Jun 3;15:4731. doi: 10.1038/s41467-024-49122-2 (PMC11148083; doi:10.1038/s41467-024-49122-2)
Supplement: Supplementary file 9 — Reporting Summary [file 41467_2024_49122_MOESM9_ESM.pdf]

nature portfolio

Corresponding author(s): Cristina Herencias, Álvaro San Millán, Jerónimo Rodríguez-Beltrán

Last updated by author(s): 16/05/2024

Reporting Summary

Nature Portfolio wishes to improve the reproducibility of the work that we publish. This form provides structure for consistency and transparency in reporting. For further information on Nature Portfolio policies, see our Editorial Policies and the Editorial Policy Checklist.

Statistics

For all statistical analyses, confirm that the following items are present in the figure legend, table legend, main text, or Methods section.

n/a	Confirmed	
☐	☒	The exact sample size (*n*) for each experimental group/condition, given as a discrete number and unit of measurement
☐	☒	A statement on whether measurements were taken from distinct samples or whether the same sample was measured repeatedly
☐	☒	The statistical test(s) used AND whether they are one- or two-sided<br>*Only common tests should be described solely by name; describe more complex techniques in the Methods section.*
☐	☒	A description of all covariates tested
☐	☒	A description of any assumptions or corrections, such as tests of normality and adjustment for multiple comparisons
☐	☒	A full description of the statistical parameters including central tendency (e.g. means) or other basic estimates (e.g. regression coefficient) AND variation (e.g. standard deviation) or associated estimates of uncertainty (e.g. confidence intervals)
☐	☒	For null hypothesis testing, the test statistic (e.g. *F*, *t*, *r*) with confidence intervals, effect sizes, degrees of freedom and *P* value noted<br>*Give P values as exact values whenever suitable.*
☒	☐	For Bayesian analysis, information on the choice of priors and Markov chain Monte Carlo settings
☒	☐	For hierarchical and complex designs, identification of the appropriate level for tests and full reporting of outcomes
☐	☒	Estimates of effect sizes (e.g. Cohen's *d*, Pearson's *r*), indicating how they were calculated

Our web collection on statistics for biologists contains articles on many of the points above.

Software and code

Policy information about availability of computer code

Data collection	Data collection: E. coli genomes were collected from the RefSeq database (https://www.ncbi.nlm.nih.gov/assembly), including all isolates from the ECOR Collection in .fna format(Ochman, H. & Selander, R. K. Standard reference strains of Escherichia coli from natural populations. J. Bacteriol. 157, 690–693 (1984).
Data analysis	As indicated in the manuscript:<br>Data sets were analyzed using R software version 4.1.2 .<br>To analyze the E. coli genomes we used EzClermont tool (https://github.com/nickp60/EzClermont)(Waters, N. R., Abram, F., Brennan, F., Holmes, A. & Pritchard, L. Easy phylotyping of Escherichia coli via the EzClermont web app and command-line tool. Access Microbiol. 2, e000143 (2020)), Mash (Ondov, B. D. et al. Mash: fast genome and metagenome distance estimation using MinHash. Genome Biol. 17, 132 (2016)) and PATO (https://github.com/irycisBioinfo/PATO) (Fernández-de-Bobadilla, M. D. et al. PATO: Pangenome Analysis Toolkit. Bioinformatics 37, 4564–4566 (2021)).<br><br>The source code used to produce the results and analyses presented in this manuscript is available from ref. 73, https://github.com/JeroRB/Collateral_Sensitivity or https://doi.org/10.5281/zenodo.11061421.

For manuscripts utilizing custom algorithms or software that are central to the research but not yet described in published literature, software must be made available to editors and reviewers. We strongly encourage code deposition in a community repository (e.g. GitHub). See the Nature Portfolio guidelines for submitting code & software for further information.

Data

Policy information about availability of data

All manuscripts must include a data availability statement. This statement should provide the following information, where applicable:

- Accession codes, unique identifiers, or web links for publicly available datasets
- A description of any restrictions on data availability
- For clinical datasets or third party data, please ensure that the statement adheres to our policy

Datasets generated and/or analysed during the current study are included in the Source Data 1 and Source Data 2 files with this paper and can be downloaded from the following repository73: https://doi.org/10.5281/zenodo.11061421. ATLAS data can be visualised through the ATLAS website (https://atlas-surveillance.com) and was downloaded from the dataset published in ref.74 Plasmid sequences can be accessed under Genbank accession numbers: PP735908, PP735911, PP735912, PP735909, PP735914, PP735913, PP735910, PP735915, and PP735916 (see also Supplementary Data 1). ECOR whole genome sequences are available under BioProject accession number PRJNA230969 [https://www.ncbi.nlm.nih.gov/bioproject/?term=PRJNA230969]

Research involving human participants, their data, or biological material

Policy information about studies with human participants or human data. See also policy information about sex, gender (identity/presentation), and sexual orientation and race, ethnicity and racism.

Reporting on sex and gender	*Use the terms sex (biological attribute) and gender (shaped by social and cultural circumstances) carefully in order to avoid confusing both terms. Indicate if findings apply to only one sex or gender; describe whether sex and gender were considered in study design; whether sex and/or gender was determined based on self-reporting or assigned and methods used. Provide in the source data disaggregated sex and gender data, where this information has been collected, and if consent has been obtained for sharing of individual-level data; provide overall numbers in this Reporting Summary. Please state if this information has not been collected. Report sex- and gender-based analyses where performed, justify reasons for lack of sex- and gender-based analysis.*
Reporting on race, ethnicity, or other socially relevant groupings	*Please specify the socially constructed or socially relevant categorization variable(s) used in your manuscript and explain why they were used. Please note that such variables should not be used as proxies for other socially constructed/relevant variables (for example, race or ethnicity should not be used as a proxy for socioeconomic status). Provide clear definitions of the relevant terms used, how they were provided (by the participants/respondents, the researchers, or third parties), and the method(s) used to classify people into the different categories (e.g. self-report, census or administrative data, social media data, etc.) Please provide details about how you controlled for confounding variables in your analyses.*
Population characteristics	*Describe the covariate-relevant population characteristics of the human research participants (e.g. age, genotypic information, past and current diagnosis and treatment categories). If you filled out the behavioural & social sciences study design questions and have nothing to add here, write "See above."*
Recruitment	*Describe how participants were recruited. Outline any potential self-selection bias or other biases that may be present and how these are likely to impact results.*
Ethics oversight	*Identify the organization(s) that approved the study protocol.*

Note that full information on the approval of the study protocol must also be provided in the manuscript.

Field-specific reporting

Please select the one below that is the best fit for your research. If you are not sure, read the appropriate sections before making your selection.

☐ Life sciences ☐ Behavioural & social sciences ☒ Ecological, evolutionary & environmental sciences

For a reference copy of the document with all sections, see nature.com/documents/nr-reporting-summary-flat.pdf

Ecological, evolutionary & environmental sciences study design

All studies must disclose on these points even when the disclosure is negative.

Study description	We investigated the collateral sensitivity response in enterobacteria associated with the acquisition of beta-lactamase carrying plasmids. We selected 7 different bla-carrying plasmids from a well-characterised collection (DelaFuente et al., 2022), 7 clinically relevant beta-lactamase genes (Rajer and Sandegren, 2022) and 9 natural E. coli strains from the ECOR collection. Our study also included the examination and analysis of MICs contained in the ATLAS database (ref 73). We designed the study to perform 6 to 9 replicates of each experiment using plasmid-free strains and beta-lactamase-free plasmids as controls.
Research sample	In this study, we focused on E. coli strains and plasmids carrying beta-lactamases of clinical origin. The plasmids were transformed or conjugated into the E. coli strains using the protocols described in the Materials and Methods section of the manuscript.

	We extended our research to enterobacteria when we examined the ATLAS database.<br>.
Sampling strategy	We selected 7 previously reported blaOXA-48-carrying plasmids (delaFuente et al., 2022) that carry different genetic mutations (deletions, insertions and single nucleotide polymorphisms (SNPs)). These mutations affect critical plasmid functions such as conjugation, antibiotic resistance or replication control.<br>We also selected 7 of the most clinically relevant β-lactamase genes (Rajer and Sandegren, 20-22).<br>Finally, we selected 9 natural E. coli strains from the ECOR collection that are representative of the E. coli phylogeny and lack known mobile β-lactamases, colistin and macrolide resistance genes (Ochman, H. & Selander, 1984).
Data collection	Experimental data were annotated in an excell file to further analysis using R software version 4.1.2.<br>ATLAS data was downloaded from the following dataset published in ref.73 (https://s3-eu-west-1.amazonaws.com/amr-prototype-data/Open+Atlas_Reuse_Data.xls)
Timing and spatial scale	For this study, we used previously characterized collections of plasmids and strains. Timing and spatial scale were referred to the corresponding studies: DelaFuente et al., 2022; Rajer and Sandegren, 2022; Ochman, H. & Selander, 1984.<br>Experimental data collection were performed from 2021 and 2023
Data exclusions	No experimental data was excluded from the analysis.
Reproducibility	We performed multiple biological replicates of each experiment (as reported in the manuscript), as indicated in each of the figure legends. All attempts to repeat the experiment were successful<br>All reported experiments were reproducible.<br>All analyses can be replicated using the source data and code provided in the manuscript.
Randomization	The covariates considered in this study were incubation temperature, culture medium and initial inoculum density. They were controlled by:<br>- Keeping all culture plates in an incubator calibrated to a constant temperature<br>- Using commercial culture media for all experiments<br>- Standardising the bacterial inoculum added to ensure that all start with the same bacterial density.<br>This approach ensures that any observed differences in bacterial growth are due to the effect of the antibiotic and minimises the influence of other variables.<br><br>Randomisation: Randomly assign Petri dishes and multi-well plates to different antibiotic treatments to ensure that any unmeasured variations in culture conditions are evenly distributed across treatment groups.
Blinding	Reading of experimental data in relation to MIC values was performed using a plate reader, and in each replicate the organisation of different strains, plasmids and beta-lactamases was modified to avoid any subjective interpretation.<br>Blinding is not applicable to the ATLAS analysis as it is a meta-analysis based study.

Did the study involve field work? ☐ Yes ☒ No

Reporting for specific materials, systems and methods

We require information from authors about some types of materials, experimental systems and methods used in many studies. Here, indicate whether each material, system or method listed is relevant to your study. If you are not sure if a list item applies to your research, read the appropriate section before selecting a response.

Materials & experimental systems

n/a	Involved in the study
☒	☐ Antibodies
☒	☐ Eukaryotic cell lines
☒	☐ Palaeontology and archaeology
☒	☐ Animals and other organisms
☒	☐ Clinical data
☒	☐ Dual use research of concern
☒	☐ Plants

Methods

n/a	Involved in the study
☒	☐ ChIP-seq
☒	☐ Flow cytometry
☒	☐ MRI-based neuroimaging

Plants

Seed stocks	*Report on the source of all seed stocks or other plant material used. If applicable, state the seed stock centre and catalogue number. If plant specimens were collected from the field, describe the collection location, date and sampling procedures.*
Novel plant genotypes	*Describe the methods by which all novel plant genotypes were produced. This includes those generated by transgenic approaches, gene editing, chemical/radiation-based mutagenesis and hybridization. For transgenic lines, describe the transformation method, the number of independent lines analyzed and the generation upon which experiments were performed. For gene-edited lines, describe the editor used, the endogenous sequence targeted for editing, the targeting guide RNA sequence (if applicable) and how the editor was applied.*
Authentication	*Describe any authentication procedures for each seed stock used or novel genotype generated. Describe any experiments used to assess the effect of a mutation and, where applicable, how potential secondary effects (e.g. second site T-DNA insertions, mosiacism, off-target gene editing) were examined.*
